# Causal effects of neuroticism on postpartum depression: a bidirectional mendelian randomization study

**DOI:** 10.1007/s00737-024-01466-w

**Published:** 2024-04-18

**Authors:** Qianying Hu, Jianhua Chen, Jingjing Ma, Yuting Li, Yifeng Xu, Chaoyan Yue, Enzhao Cong

**Affiliations:** 1 Shanghai Mental Health Center, Shanghai Jiao Tong University School of Medicine, 600 South Wanping Road, Shanghai, 200030 China; 2 School of Medicine, Shanghai Tenth People’s Hospital, Tongji University, Shanghai, China; 3 Obstetrics and Gynecology Hospital of Fudan University, Fang Xie Road, No419, Shanghai, Shanghai China

**Keywords:** Postpartum depression, Neuroticism, Mendelian randomization, Genetic cause

## Abstract

**Purpose:**

Postpartum depression (PPD) brings adverse and serious consequences to both new parents and newborns. Neuroticism affects PPD, which remains controversial for confounding factors and reverse causality in cross-sectional research. Therefore, mendelian randomization (MR) study has been adopted to investigate their causal relationship.

**Methods:**

This study utilized large-scale genome-wide association study genetic pooled data from three major databases: the United Kingdom Biobank, the European Bioinformatics Institute, and the FinnGen databases. The causal analysis methods used inverse variance weighting (IVW). The weighted median, MR-Egger method, MR-PRESSO test, and the leave-one-out sensitivity test have been used to examine the results’ robustness, heterogeneity, and horizontal pleiotropy. The fixed effect model yielded the results of meta-analysis.

**Results:**

In the IVW model, a meta-analysis of the MR study showed that neuroticism increased the risk of PPD (OR, 1.17; 95% CI, 1.11–1.25, *p* < 0.01). Reverse analysis showed that PPD could not genetically predict neuroticism. There was no significant heterogeneity or horizontal pleiotropy bias in this result.

**Conclusion:**

Our study suggests neuroticism is the risk factor for PPD from a gene perspective and PPD is not the risk factor for neuroticism. This finding may provide new insights into prevention and intervention strategies for PPD according to early detection of neuroticism.

**Supplementary Information:**

The online version contains supplementary material available at 10.1007/s00737-024-01466-w.

## Introduction

Postpartum depression (PPD) refers to a specifier for depressive disorders meeting the Diagnostic and Statistical Manual of Mental Disorders, Fifth Edition, in the weeks or months following delivery (American Psychiatric Association, 2013). The prevalence of PDD falls in the 12-26% range (Liu et al. 2022; O’Hara and McCabe 2013; Shorey et al. 2018). Generally, economically developed countries tend to exhibit a lower incidence of PPD (Escribà-Agüir and Artazcoz 2011; Howard et al. 2014; Shorey et al. 2018). It is worth noting that even new fathers may experience PPD at a rate of about 8.4% (Cameron et al. 2016). The main negative consequences of PDD include harming the mother’s physical and psychological health, impacting her social and intimate relationships, giving rise to her risky and maladaptive behaviors, affecting the infant’s growth and development, and influencing the mother-infant relationship (Slomian et al. 2019). Meanwhile, PPD poses a risk for the subsequent development of bipolar disorder and may also confuse the detection and treatment of postpartum bipolar disorders (Sharma et al. 2009). The literature has indicated numerous risk factors for PDD, such as the mother’s medical conditions and delivery-related factors (e.g., gestational diabetes mellitus,

gestational diabetes, vitamin D deficiency, obese and overweight, cesarean section, multiple births, preterm and low birth-weight infants, negative birth experience, clinical delivery difficulties, postpartum anemia), the mother’s mental health and psychological factors (e.g., history of depression before delivery, postpartum sleep disruption, lack of social support, level of prenatal attachment to child, quality of romantic relationship, first-time mothers, woman’s age), and social and environmental factors (e.g., violence and abuse, immigration status, traditional dietary pattern) (Bradshaw et al. 2022; Liu et al. 2022; Smorti et al. 2019; Zhao and Zhang 2020). In addition to the factors mentioned above, personality traits, especially neuroticism, may increase the risk of PPD (MartÍN-Santos et al. 2012).

Neuroticism, as a personality trait, manifests in heightened emotional sensitivity. Individuals with high neuroticism scores are more susceptible to feelings of anxiety, worry, fear, anger, frustration, envy, jealousy, guilt, depression, and loneliness (Jeronimus et al. 2016). In scientific research, personality measurement involving neuroticism is often operationalized and standardized by questionnaires, such as the Eysenck Personality Questionnaire (Eysenck et al. 1985), NEO Personality Inventory (Costa and McCrae 1992), and Big Five Inventory (Soto and John 2009). Among the three personality traits—extraversion, neuroticism, and psychoticism—only neuroticism can predict the occurrence of postpartum depression (MartÍN-Santos et al. 2012). Neuroticism was linked to a single nucleotide polymorphism in the hydroxysteroid (11-beta) dehydrogenase 1 gene, mediating its association with postpartum depression (Iliadis et al. 2017). For maternal depression, expression levels of hydroxysteroid (11-beta) dehydrogenase positively correlate with serotonin transporter (Ponder et al. 2011). Serotonin transporter may influence the relationship between neuroticism and PPD (Canli and Lesch 2007; Shapiro et al. 2012; Takano et al. 2007).

Some studies indeed suggested a correlation between neuroticism and postpartum depression (Axfors et al. 2017; Boyce et al. 1991), while others indicate that neuroticism is associated with postpartum depression within the first 3–5 days but not after 6–9 weeks postpartum (Iliadis et al. 2015; Imširagić et al. 2014). One study reports that within the first week after delivery, neuroticism transitions from a significant risk factor to a non-significant one after full adjustment (Maliszewska et al. 2016). Therefore, the uncertain relationship between neuroticism and postpartum depression requires further validation. Additionally, it remains uncertain whether neuroticism has an influence on postpartum depression predominantly from biological factors, or just by socio-psychological influences. This study adopted a Mendelian randomization study to address these problems.

The Mendelian randomization (MR) posits that parental alleles are randomly distributed to offspring during gamete formation. If the genotype determines the phenotype and the genotype is associated with the disease through the phenotype, then the genotype can serve as an instrumental variable to infer the association between the phenotype and the disease (Bowden & Holmes, 2019). MR studies utilize genetic variation as an instrumental variable to assess potential causal relationships between exposure variables and outcome variables, aiming to reduce potential biases caused by confounding and reverse causation (Skrivankova et al. 2021). Therefore, this study employs an MR study to investigate the causal association between neuroticism and postpartum depression, overcoming the limitations of observational studies. We hypothesize that neuroticism genetically increases the risk of postpartum depression and can predict its occurrence.

## Methods

### Study design

An MR study design delineated a causal relationship between neuroticism and PPD. Within this methodological framework, single nucleotide polymorphisms (SNPs) associated with neuroticism were utilized as instrumental variables representing the exposures, with PPD being the outcome (Bowden et al. 2017). Three assumptions were elucidated for MR analyses as follows: (1) A direct correlation exists between the SNPs, serving as instrumental variables, and neuroticism as the exposures; (2) Confounding variables remain independent of the association between SNPs and neuroticism; And (3) the causal pathway linking SNPs to the outcomes (PPD) is only through neuroticism.

### Data sources and filter instrumental variables

Neuroticism data sources were sourced from the United Kingdom Biobank (UKB) and the European Bioinformatics Institute (EBI), encompassing the Neuroticism score (id: ukb − b−4630), Neuroticism (id: ebi − a−GCST005232), and Neuroticism score (id: ukb − a−230). Due to the bidirectional causal testing design of the study, the genetic variant-exposure and genetic variant-outcome should come from different samples (Skrivankova et al. 2021). ,Therefore, we acquired the SNPs of postpartum depression from FinnGen (id: finn-b-O15_POSTPART_DEPR). Detailed characteristics of the data sources are presented in Table 1.

**Table 1:** The characteristics of data sources used

Traits	Datasets	Unit	Population	Sample size	Number of SNPs	instrumental variable	Consortium	Year
The Neuroticism score	UKB-b-44,630	SD	European	374,323	9,851,867	108	MRC-IEU	2018
The Neuroticism	EBI-a-GCST005232	UA	European	329,821	18,436,568	65	Neale Lab	2017
The Neuroticism score	UKB-a-230	SD	European	274,108	10,894,596	62	Neale Lab	2017
Postpartum depression	FINN-b-O15_POSTPART_DEPR	UA	European	66,665	16,376,275	11	UA	2021

SD: Standard deviation

UA: Unavailable

SNP: Single nucleotide polymorphisms

Initially, single nucleotide polymorphisms (SNPs) associated exposures meeting a significance level of *P*-value ≤ 5 × 10^–8^ were extracted. The independence of included SNPs as instrumental variables (IVs) was assured by utilizing a clumping method with a linkage disequilibrium threshold of r^2^ > 0.001 within a 10,000 kb window. The F-statistics (F = beta^2^/se*2*) for all IVs, indicating their predictive capability, were required to be higher than 10. SNPs significantly associated with the exposure, and simultaneously associated with the outcome (with a *p*-value < 5 × 10^–5^) were excluded. These specific thresholds of summary-level statistics of the association of each genetic variant are required by the Two-sample MR approach (Georgakis et al. 2019). Subsequently, these SNPs were harmonized and filtered using the Setiger filtering method. Supplementary File 1 provides information on the remaining SNPs.

### Data analysis

The “Two Sample MR” package in R (version 4.3.0) was employed for Mendelian randomization analysis. The primary method for assessing the causal effects of neuroticism on postpartum depression was the inverse-variance-weighted (IVW) (Bowden et al. 2015), The weighted-median estimator, weighted mode, and MR-Egger regression were applied to gauge the robustness of the IVW results(Burgess and Thompson 2017). The heterogeneity of the included results was analyzed with a significance level set at *p* = 0.1. If *p* ≥ 0.1, indicating no statistically significant heterogeneity among the results of IVW, a fixed effect model was applied for meta-analysis. If *p* < 0.1, suggesting statistical heterogeneity among the study outcomes, a random effect model was used for meta-analysis (Riley et al. 2011). To investigate reverse causation, a bidirectional study design was adopted. Heterogeneity among results was assessed using the Cochran-Q statistic test(Bowden et al. 2016). To address potential horizontal pleiotropy, the MR-Egger intercept and MR PRESSO methods were implemented (Burgess and Thompson 2017). Funnel plots were generated to visualize the individual Wald ratios for each SNP plotted against their precision.

## Results

We explored the SNPs for neuroticism by identifying them in three datasets and for postpartum depression in one dataset as shown in Table 1. We found 108, 65, and 66 SNPs for neuroticism respectively (*p* < 5 × 10^–8^, R^2^ < 0.001 and > 10,000 kb, F > 10). The details of SNPs associated with both the exposures and outcome GWASs are provided in Supplementary File 1.

**Fig. 1:**
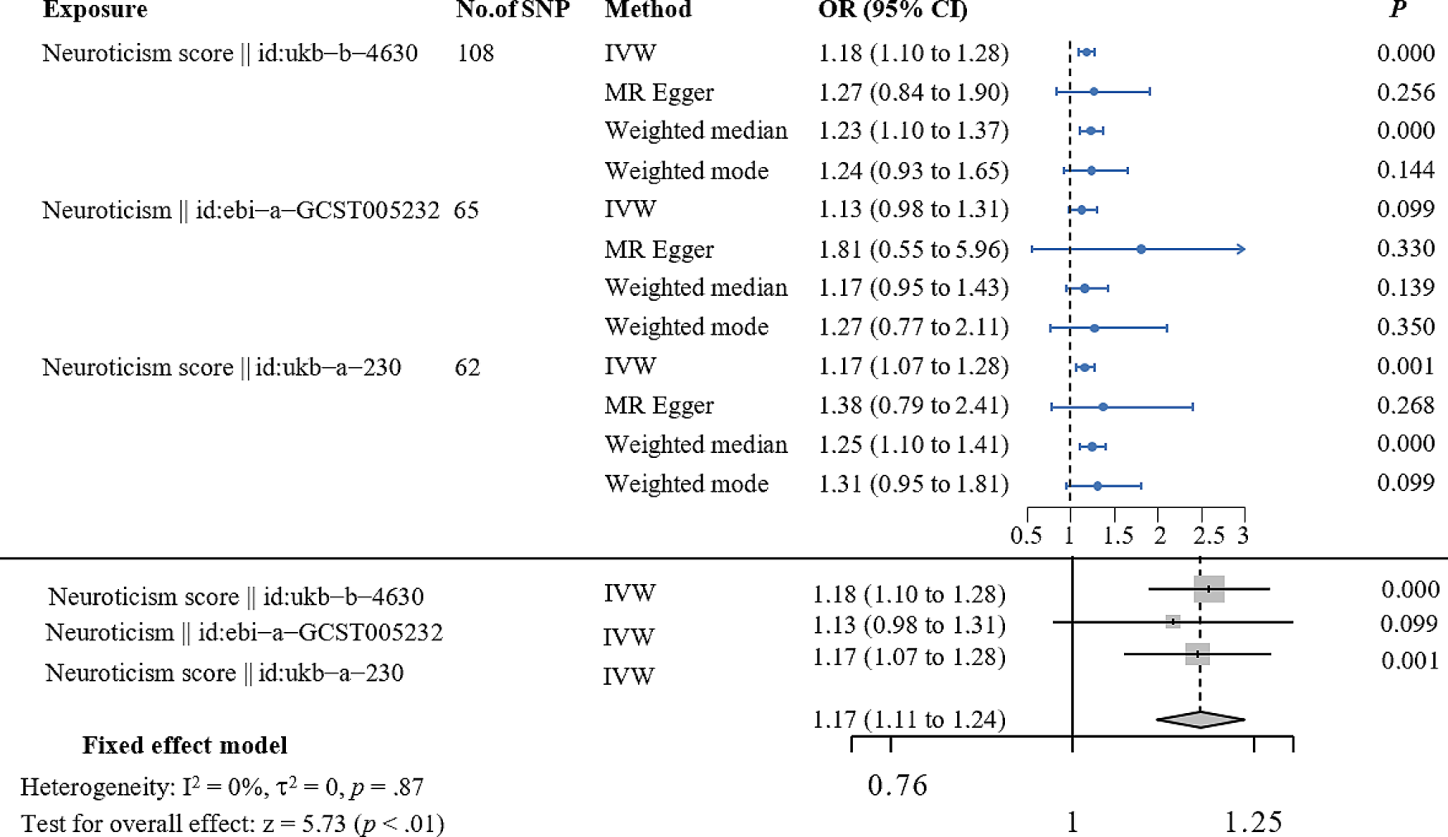
A meta-analysis of Mendelian randomization results about neuroticism on risk of postpartum depression

**Fig. 2:**
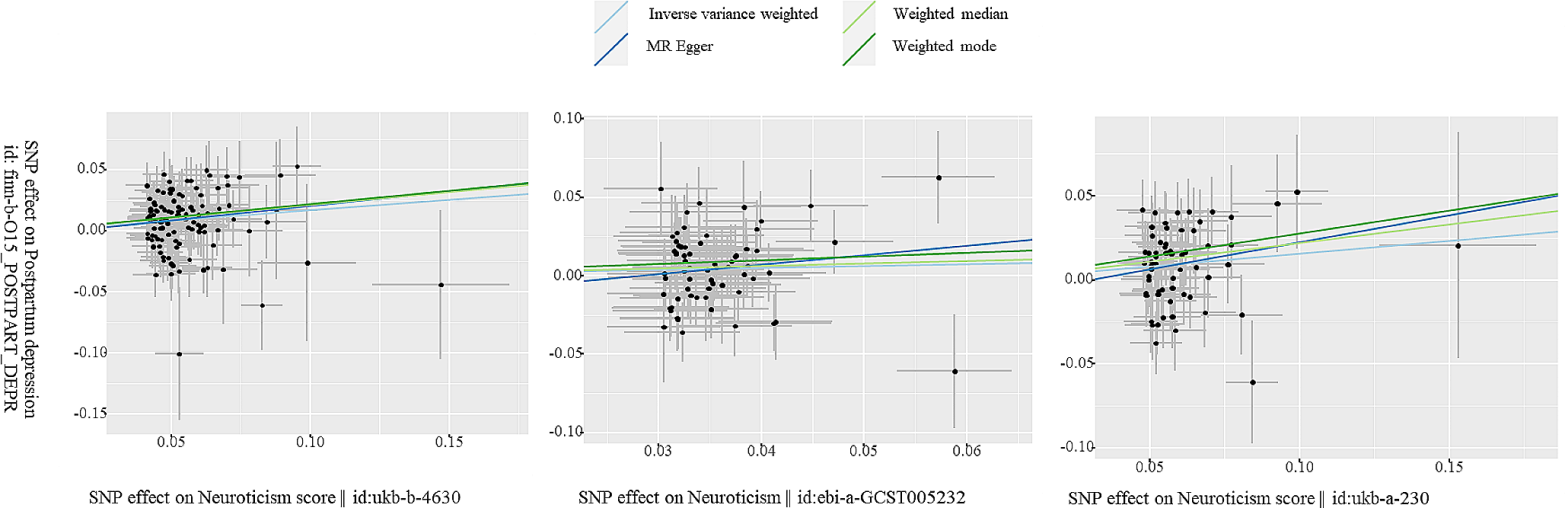
Scatter plots of Mendelian randomization analyses

A meta-analysis of MR results about neuroticism on the risk of postpartum depression was shown in Fig. 1, in which neuroticism genetically predicted postpartum depression with an odds ratio of 1.17 (95% CI, 1.11–1.24). Furthermore, the meta-analysis model exhibited no heterogeneity (*p* = 0.87) and a good overall effect (*p* < 0.01). In detail, the odds ratio (OR) of the Neuroticism score (id: ukb − b−4630) for PPD was 1.18 (95%CI, 1.10–1.28; *p* = 0.000) in the IVW MR analysis. Similar results were observed in the Neuroticism score (id: ukb − a−230) (OR:1.17, 95%CI, 1.07–1.28, *p* = 0.001]. Neuroticism (id: ebi − a−GCST005232) was marginally significantly associated with PPD using the IVW method, with an odds ratio of 1.13 (95% CI 0.98–1.31; *p* = 0.099). The other methods including MR-Egger, Weighted median, and Weighted mode examine the robustness of the IVW method. We found all predictive directions are consistent, but there is heterogeneity in significance. A scatter plot was used to visualize the causal effect of neuroticism on the risk of PPD shown in Fig. 2. The results of the reverse MR analyses indicated that PPD has no causal effect on neuroticism (Table 2).

**Table 2:** The reverse Mendelian randomization results of postpartum depression on risk of Neuroticism

Exposure. id	NO. SNPs	Outcomes. id	OR (95%CI)	P
postpartum depression (id: finn-b-O15_POSTPART_DEPR)	11	The Neuroticism score (id: UKB-b-44,630)		
		Inverse variance weighted	0.98 (0.91, 1.04)	0.47
		MR Egger	0.97 (0.70, 1.35)	0.86
		Weighted median	0.94 (0.87, 1.02)	0.14
		Weighted mode	0.93 (0.82, 1.04)	0.23
		The Neuroticism (id: ebi-a-GCST005232)		
		Inverse variance weighted	1.01 (0.98, 1.03)	0.61
		MR Egger	1.00 (0.92, 1.09)	0.97
		Weighted median	1.00 (0.96, 1.03)	0.85
		Weighted mode	0.99 (0.95, 1.04)	0.70
		The Neuroticism score (id: ukb-a-230)		
		Inverse variance weighted	1.01 (0.94, 1.09)	0.74
		MR Egger	0.97 (0.68, 1.40)	0.88
		Weighted median	0.95 (0.87, 1.04)	0.27
		Weighted mode	0.93 (0.81, 1.07)	0.35

NO. SNPs: Number of single nucleotide polymorphisms

OR: Odds ration

CI: Confidence interval

Table 3 shows the results of the Cochran’s Q statistic test. There was no significant heterogeneity in SNP effects (All *p* > 0.05). To investigate the direction of the horizontal pleiotropy, MR-Egger intercepts, and MR-PRESSO were utilized. The results of MR-Egger intercept tests did not show any significant horizontal pleiotropy bias (All *p* > 0.05) as shown in Table 3.

**Table 3:** The analysis of heterogeneity and horizontal pleiotropy in the risk of neuroticism on postpartum depression

Exposures (id)	NO. SNPs	Cochrane’s Q				Pleiotropy	
		MR-Egger		IVW		MR-Egger	MR-PRESSO
		Q	P	Q	P	P	P
The Neuroticism score (UKB-b-44,630)	108	109.70	0.38	109.81	0.40	0.74	0.41
The Neuroticism (evi-a-GCST005232)	65	68.93	0.28	69.60	0.30	0.44	0.30
The Neuroticism score (ukb-b-230)	62	68.82	0.20	69.21	0.22	0.60	0.24

NO. SNPs: Number of single nucleotide polymorphisms

IVW: Inverse variance weighted

## Discussion

This study has revealed that neuroticism increases the risk of postpartum depression (PPD) from a genetic perspective, but PPD cannot predict the risk of neuroticism. Our results are consistent with previous observational research. A total of 1,974 women without any mental disorders during pregnancy participated in a 32-week longitudinal study within 2–3 days postpartum. The findings indicated that women experiencing postpartum depressive symptoms or PPD scored significantly higher in neuroticism compared to the healthy control group (MartÍN-Santos et al. 2012). Furthermore, a meta-analysis suggested that neuroticism increases the risk of PPD by 1.37 folds (Puyané et al. 2022). However, reverse Mendelian randomization (MR) results indicate that PPD does not lead to neuroticism. This is because neuroticism has its unique genetic loci, specific tissue expression, and cell expression (Nagel et al. 2018), which interacted with environmental factors, leading to a stable personality trait over time (Barlow et al. 2014), rather than being the outcome of PPD occurring within approximately four weeks after childbirth.

Genetical mechanisms and sociological data affirm neuroticism as an independent risk factor for postpartum depression, rather than a mediator for traumatic influence. Existing literature reports that postpartum anxiety fully mediates the relationship between neuroticism and depressive symptoms assessed two weeks postpartum, and this mediating effect is also influenced by the mode of delivery (Roman et al. 2019). Specifically, mothers with high levels of neuroticism also reported elevated postpartum anxiety levels 3 to 4 days after childbirth, which is associated with postpartum depression. This association may be attributed to anxiety partially stemming from environmental control, with neurotic personality traits making individuals more susceptible to a loss of environmental control, ultimately leading to postpartum depression(Gross and Hen 2004). This meditation model is exclusively in women undergoing cesarean section, possibly because women opting for this procedure without specific medical indications may experience higher anxiety, lack of confidence, and fear of childbirth (Olieman et al. 2017).

The genetic loci associated with neuroticism contain genes related to depression, primarily distributed on chromosomes 2 and 19 genetic loci (Nagel et al. 2018). Extensive functional annotations of neuroticism include the neurogenesis pathway, which would be impaired by Postpartum estrogen withdrawal (Nagel et al. 2018; Zhang et al. 2016). Meanwhile, the neural circuit activity of GABAergic neurons projecting to serotonergic neurons may decrease due to ovarian hormone withdrawal, leading to the occurrence of depression, while serotonergic neurons are involved in the genetic correlation of neuroticism, implicating the involvement of specific cell types (Nagel et al. 2018; Tao et al. 2023).

The serotonin transporter serves as an important mediator between neuroticism and postpartum depression. Serotonin transporter binding is positively correlated with neuroticism score (Takano et al. 2007). Abnormalities in genes encoding serotonin transporter are associated with genes for emotional dysregulation (Canli and Lesch 2007), leading to the vulnerability of depression. Neuroticism at age 25 is linked to increased emotional dysregulation at age 36 (Kokkonen and Pulkkinen 2001). The impact of neuroticism, particularly on emotional stability circuits such as the prefrontal cortex, constitutes an independent pathological mechanism. Neuroimaging reveals a significant negative association between neuroticism scores and prefrontal cortex activity during negative emotion processing (Yang et al. 2020). Proteomics analysis of the prefrontal cortex in depression also indicates anomalies (Johnston-Wilson et al. 2000). Additionally, the menstrual cycle influences emotional processing in the prefrontal cortex (Protopopescu et al. 2005). All of them suggest that the prefrontal cortex may be a critical brain region governing the predictive role of neuroticism on postpartum depression. Clinical evidence indicated that the prefrontal cortex could serve as a potential target for transcranial direct current stimulation in the treatment of postpartum depression(Ironside et al. 2016).

## Conclusion

Neuroticism represents an independent risk factor for postpartum depression, as confirmed by genetic research. The causal relationship established in this study further underscores the value of early detection for the population with neuroticism and then intervention, which emphasizes the importance of preventing postpartum depression.

### Limitation

In the robustness analysis of the IVW method, MR-Egger and weighted mode showed insufficient as non-significant results. The statistical power of MR-Egger is compromised by outliers resulting from measurement errors. Even with the removal of outliers, measurement errors in other variables may still contribute to heterogeneity in the results. Once MR-Egger violates the no measurement error assumption, the bias it introduces tends to be greater than that of the IVW estimate, and it is particularly susceptible to the influence of weak instrument bias (Bowden et al., 2016). The weighted mode method is sensitive for nonlinear effects, leading to biased results (Hartwig et al. 2017). However the weighted median method supports the robustness of IVW. In addition, we conducted outlier detection and removal strategies to outlier detection and removal strategies (Bowden & Holmes, 2019). Supplementary File 2 shows the good robustness of IVW. Additionally, a limitation arises in this study when incorporating exposure variables, as one trait is the neuroticism score rather than neuroticism, potentially leading to inconsistencies in the instrumental variables. Moreover, in the process of obtaining samples for this study, data were acquired through public data resources without access to detailed reports from relevant databases. This lack of detailed information may result in the inability to account for some confounding factors, potentially impacting the interpretation and generalizability of the study results.

### Implications

This study has genetically confirmed neuroticism as a significant risk factor for postpartum depression, demonstrating its predictive ability for postpartum depressive tendencies. These findings offer new insights into the prevention of postpartum depression. Before childbirth, the risk of developing postpartum depression can be assessed through personality trait testing. If a pregnant woman exhibits a higher neuroticism score, caution should be exercised regarding the potential development of postpartum depression. Additionally, in the treatment of postpartum depression, tailored treatment plans can be developed based on personality characteristics to enhance the individual’s response to the treatment. Lastly, neuroticism may serve as a crucial distinguishing indicator for subtypes of postpartum depression, offering clear markers for a more accurate classification of postpartum depression.

## Electronic supplementary material

Below is the link to the electronic supplementary material.

Supplementary Material 1

Supplementary Material 2
