# Supplementary material for: Causal effects of neuroticism on postpartum depression: a bidirectional mendelian randomization study
Source: Arch Womens Ment Health. 2024 Apr 18;27(5):837–44. doi: 10.1007/s00737-024-01466-w (PMC11405473; doi:10.1007/s00737-024-01466-w)
Supplement: Supplementary file 1 — Supplementary Material 1 [file 737_2024_1466_MOESM1_ESM.docx]

**Causal Effects of Neuroticism on Postpartum Depression: A Bidirectional Mendelian Randomization Study**

**Acknowledgments**

We express our gratitude to the United Kingdom Biobank and European Bioinformatics Institute for generously sharing GWAS pooled data with neuroticism datasets (id: ukb−b−4630 and ukb−a−230, id: ebi−a−GCST005232). In addition, we extend our appreciation to the FinnGen study for providing valuable GWAS pooled data on postpartum depression (id: finn-b-O15_POSTPART_DEPR) included in this research.

**Ethical Statement**

The present Mendelian randomization analysis relies on pooled data, and ethical approval has been duly obtained. It is important to note that secondary analyses of pooled data do not necessitate separate ethical approval.

**Funding**

The study was funded by the Shanghai Planning Office of Philosophy and Social Science, Grant Number: 2019BSH012. The funders had no role in study design, data collection, data analysis, data interpretation, or writing of the manuscript.

**Author contribution**

Enzhao Cong, Yifeng Xu, Chaoyan Yue: Conceptualization, Data curation, Formal analysis, Funding acquisition, Methodology, Project administration, Resources, Software, Supervision, Validation, Visualization and Writing - review & editing.

Qianying Hu: Conceptualization, Project administration, Validation, Visualization, Writing - original draft, and Writing - review & editing.

Jianhua Chen, Jingjing Ma, Luting Li: Methodology, Project administration, Resources, Writing - review & editing

**Conflicts of Interest**

All authors declare no competing interests.

**Availability of data**

Data and materials will be available from https://gwas.mrcieu.ac.uk/(id: ukb−b−4630 and ukb−a−230, id: ebi−a−GCST005232 and finn-b-O15_POSTPART_DEPR respectively).
