# Supplementary material for: Causal effects of neuroticism on postpartum depression: a bidirectional mendelian randomization study
Source: Arch Womens Ment Health. 2024 Apr 18;27(5):837–44. doi: 10.1007/s00737-024-01466-w (PMC11405473; doi:10.1007/s00737-024-01466-w)
Supplement: Supplementary file 2 — Supplementary Material 2 [file 737_2024_1466_MOESM2_ESM.docx]

Dear Reviewers and Editors:

Thank you very much for taking the time to review our manuscript as well as for your valuable comments. We greatly appreciate your insightful feedback, which has helped to enhance the quality of our work. We carefully reviewed each of your comments and have made revisions to our manuscript accordingly. Please find our responses to your comments below, and let us know if any parts require further clarification.

We appreciate your continued consideration and hope that our revised manuscript meets your expectations. Thank you again for your kind review.

Reviewer #2: Thank you for the opportunity to review the manuscript entitled "Causal Effects of Neuroticism on Postpartum Depression: A Bidirectional Mendelian Randomization Study".

This manuscript presents a study aimed at investigating the causal association between neuroticism and Postpartum depression (PPD). This is an important topic to study since PPD, especially if untreated, increases the risk of a multitude of serious long-term consequences for the woman, her child, and their bond.

I think this topic is worth investigating, and the authors provided a novel contribution to the literature.

However, before of full acceptance, I would like the authors to address some important points.

Introduction

Overall, I found this section a little lacking and sometimes not properly organized. I understand that they are not the center of the manuscript, but it would be useful if the authors would spend more words in talking about why it is important to deepen the theme of PPD (consequences, prevalence, etc.) also referring to the most recent literature. This would allow a better understanding of why the study conducted is important. In addition, the authors should stress more why it is important to investigate the causal association between neuroticism and PPD.

Therefore, I highlight below some issues:

1)	p. 1, lines 35-37: the authors should refer to the DSM 5 (or even the DSM 5-TR) and not to the DSM-IV when talking about PPD, also reporting the reference.

Thank you very much for your suggestion. Following your advice, we have updated the reference and the definition of PPD.

Revision: Lines 35-37

Postpartum depression (PPD) refers to a specifier for depressive disorders meeting the Diagnostic and Statistical Manual of Mental Disorders, Fifth Edition, in the weeks or months following delivery (American Psychiatric Association, 2013).

American Psychiatric Association, D. (2013). *Diagnostic and statistical manual of mental disorders: DSM-5* (Vol. 5): American psychiatric association Washington, DC.

2)	p. 1, lines 36-37: Authors should refer to more recent studies regarding the prevalence of PPD, such as (but not limited to):

a.	Shorey, S., Chee, C. Y. I., Ng, E. D., Chan, Y. H., San Tam, W. W., & Chong, Y. S. (2018). Prevalence and incidence of postpartum depression among healthy mothers: A systematic review and meta-analysis. Journal of Psychiatric Research, 104, 235-248.

b.	Liu, X., Wang, S., & Wang, G. (2022). Prevalence and risk factors of postpartum depression in women: a systematic review and meta‐analysis. Journal of Clinical Nursing, 31(19-20), 2665-2677.

Thank you very much for your elaborative suggestion. Following your advice, we have updated the references and the prevalence of PPD.

Revision: Lines 37-39

The prevalence of PDD falls in the 12%-26% range (Liu et al., 2022; O'Hara & McCabe, 2013; Shorey et al., 2018).

Shorey, S., Chee, C. Y. I., Ng, E. D., Chan, Y. H., San Tam, W. W., & Chong, Y. S. (2018). Prevalence and incidence of postpartum depression among healthy mothers: A systematic review and meta-analysis. Journal of Psychiatric Research, 104, 235-248.

Liu, X., Wang, S., & Wang, G. (2022). Prevalence and risk factors of postpartum depression in women: a systematic review and meta‐analysis. Journal of Clinical Nursing, 31(19-20), 2665-2677.

O'Hara, M. W., & McCabe, J. E. 2013. Postpartum depression: current status and future directions. Annu Rev Clin Psychol, 9, 379-407. doi:10.1146/annurev-clinpsy-050212-185612

3)	p. 1, lines 35-37: Why did the authors report the rate of PPD in fathers?

Thanks for your question. We included this information to provide a comprehensive understanding of mental health outcomes within the context of PDD. While PPD is commonly associated with mothers, the research has acknowledged its occurrence in fathers as well. By including the rate of PPD in fathers, we aimed to offer insights into the broader impact of parenthood on mental health and to contribute to the evolving understanding of PPD beyond maternal experiences. We appreciate the opportunity to clarify this aspect of our research. If any areas are inappropriate, we will proceed with further modifications.

4)	pp. 1-2, lines 44-46: Authors should deal more with the consequences of PPD by referring to more recent studies (for example Slomian, J., Honvo, G., Emonts, P., Reginster, J. Y., & Bruyère, O. (2019). Consequences of maternal postpartum depression: A systematic review of maternal and infant outcomes. Women's Health, 15, 1745506519844044.)

Thank you very much for your suggestion. Following your advice, we have updated the consequences of PPD. As the literature provided covers the consequences of PPD as previously listed, we have removed the earlier content.

Revision: Lines 42-46

The main negative consequences of PDD include harming the mother's physical and psychological health, impacting her social and intimate relationships, giving rise to her risky and maladaptive behaviors, affecting the infant's growth and development, and influencing the mother-infant relationship (Slomian et al., 2019).

Slomian, J., Honvo, G., Emonts, P., Reginster, J. Y., & Bruyère, O. (2019). Consequences of maternal postpartum depression: A systematic review of maternal and infant outcomes. Women's Health, 15, 1745506519844044.

5)	p. 2, lines 46-49: The authors - in addition to referring to the most recent literature regarding the risk factor for PPD - should explain in more detail why it is important to investigate neuroticism as a risk factor that can increase the risk of PPD. This would help the reader to better understand the logic behind the study. Some studies that authors might consider are:

a.	Liu, X., Wang, S., & Wang, G. (2022). Prevalence and risk factors of postpartum depression in women: a systematic review and meta‐analysis. Journal of Clinical Nursing, 31(19-20), 2665-2677.

b.	Zhao, X. H., & Zhang, Z. H. (2020). Asian Journal of Psychiatry, 53, 102353.

c.	Puyané, M., Subirà, S., Torres, A., Roca, A., Garcia-Esteve, L., & Gelabert, E. (2022). Personality traits as a risk factor for postpartum depression: A systematic review and meta-analysis. Journal of Affective Disorders, 298, 577-589.

d.	Bradshaw, H., Riddle, J. N., Salimgaraev, R., Zhaunova, L., & Payne, J. L. (2022). Risk factors associated with postpartum depressive symptoms: A multinational study. Journal of Affective Disorders, 301, 345-351.

e.	Smorti, M., Ponti, L., & Pancetti, F. (2019). A comprehensive analysis of post-partum depression risk factors: the role of socio-demographic, individual, relational, and delivery characteristics. Frontiers in Public Health, 7, 295.

We appreciate your insightful suggestion and the references you have provided on this matter. We have integrated the content, and the revised version is as follows:

Revision: Lines 53-62

The literature has indicated numerous risk factors for PDD, such as the mother’s medical conditions and delivery-related factors (e.g., gestational diabetes mellitus,

gestational diabetes, vitamin D deficiency, obese and overweight, cesarean section, multiple births, preterm and low birth-weight infants, negative birth experience, clinical delivery difficulties, postpartum anemia), the mother’s mental health and psychological factors (e.g., history of depression before delivery, postpartum sleep disruption, lack of social support, level of prenatal attachment to child, quality of romantic relationship, first-time mothers, woman’s age), and social and environmental factors (e.g., violence and abuse, immigration status, traditional dietary pattern) (Bradshaw et al., 2022; Liu et al., 2022; Smorti et al., 2019; Zhao & Zhang, 2020).

Liu, X., Wang, S., & Wang, G. (2022). Prevalence and risk factors of postpartum depression in women: a systematic review and meta‐analysis. Journal of Clinical Nursing, 31(19-20), 2665-2677.

Zhao, X. H., & Zhang, Z. H. 2020. Risk factors for postpartum depression: An evidence-based systematic review of systematic reviews and meta-analyses. Asian J Psychiatr, 53, 102353. doi:10.1016/j.ajp.2020.102353

Bradshaw, H., Riddle, J. N., Salimgaraev, R., Zhaunova, L., & Payne, J. L. (2022). Risk factors associated with postpartum depressive symptoms: A multinational study. Journal of Affective Disorders, 301, 345-351.

Smorti, M., Ponti, L., & Pancetti, F. (2019). A comprehensive analysis of post-partum depression risk factors: the role of socio-demographic, individual, relational, and delivery characteristics. Frontiers in Public Health, 7, 295.

Discussion

In this section, the authors refer to many aspects that were not taken into consideration in the introduction section (e.g., Genetic mechanisms, serotonin transporter, Proteomics analysis). Since they appear to be important for explaining the results of the study, the authors should introduce these aspects in the introduction. This would make it possible to give greater robustness to the study and to better link what was written in the entire manuscript.

We appreciate the reviewer's constructive feedback regarding the need for better integration of certain aspects discussed in the results section into the introduction section of our manuscript.

Revision: Lines 53-62

Neuroticism was linked to a single nucleotide polymorphism in the hydroxysteroid (11-beta) dehydrogenase 1 gene, mediating its association with postpartum depression (Iliadis et al., 2017). For maternal depression, expression levels of hydroxysteroid (11-beta) dehydrogenase positively correlate with serotonin transporter (Ponder et al., 2011). Serotonin transporter may influence the relationship between neuroticism and PPD (Canli & Lesch, 2007; Shapiro et al., 2012; Takano et al., 2007).

Iliadis, S. I., Comasco, E., Hellgren, C., Kollia, N., Sundström Poromaa, I., & Skalkidou, A. 2017. Associations between a polymorphism in the hydroxysteroid (11-beta) dehydrogenase 1 gene, neuroticism, and postpartum depression. J Affect Disord, 207, 141-147. doi:10.1016/j.jad.2016.09.030

Ponder, K. L., Salisbury, A., McGonnigal, B., Laliberte, A., Lester, B., & Padbury, J. F. 2011. Maternal depression and anxiety are associated with altered gene expression in the human placenta without modification by antidepressant use: implications for fetal programming. Dev Psychobiol, 53(7), 711-723. doi:10.1002/dev.20549

Canli, T., & Lesch, K. P. 2007. Long story short: the serotonin transporter in emotion regulation and social cognition. Nat Neurosci, 10(9), 1103-1109. doi:10.1038/nn1964

Shapiro, G. D., Fraser, W. D., & Séguin, J. R. 2012. Emerging risk factors for postpartum depression: serotonin transporter genotype and omega-3 fatty acid status. Can J Psychiatry, 57(11), 704-712. doi:10.1177/070674371205701108

Takano, A., Arakawa, R., Hayashi, M., Takahashi, H., Ito, H., & Suhara, T. 2007. Relationship Between Neuroticism Personality Trait and Serotonin Transporter Binding. Biological Psychiatry, 62(6), 588-592.

Reviewer #3 This study adopts the Mendelian randomization study to investigate the causal relationship between neuroticism and postpartum depression. The research design is rigorous, the data analysis is comprehensive, and the conclusions drawn are scientifically and reliably supported. The study is significant in understanding the relationship between neuroticism and PPD and in developing targeted prevention and intervention strategies, especially from the gene perspective. However, there are still some areas that require further elaboration and expansion by the authors.

1. The author employed three databases related to neuroticism. Have other databases been considered as well? How representative are the samples?

Thanks for your question. The databases we selected are among the most authoritative and have large sample sizes in current whole-genome analyses. For more details, please refer to the following website links (the United Kingdom Biobank: https://gwas.mrcieu.ac.uk/, the European Bioinformatics Institute: https://biobank.ndph.ox.ac.uk/ukb/, and the FinnGen databases: https://r10.finngen.fi/). During our literature review, we also found that databases containing SNPs related to neuroticism typically originate from the databases we utilized. Please refer to the listed references. Therefore, the databases used in this study are representative and of high quality.

Nagel, M., Jansen, P. R., Stringer, S., Watanabe, K., de Leeuw, C. A., Bryois, J., Savage, J. E., Hammerschlag, A. R., Skene, N. G., Muñoz-Manchado, A. B., 23andMe Research Team, White, T., Tiemeier, H., Linnarsson, S., Hjerling-Leffler, J., Polderman, T. J. C., Sullivan, P. F., van der Sluis, S., & Posthuma, D. (2018). Meta-analysis of genome-wide association studies for neuroticism in 449,484 individuals identifies novel genetic loci and pathways. Nature Genetics, 50(7), 920–927. https://doi.org/10.1038/s41588-018-0151-7IF: 30.8 Q1

Ardissino, M., Geddes-Barton, M., & Banerjee, A. (2024). Genetically predicted body mass index and maternal outcomes of pregnancy: A two-sample Mendelian randomization study. BJOG: an international journal of obstetrics and gynecology, 131(4), 493–499. https://doi.org/10.1111/1471-0528.17650IF: 5.8 Q1

Park, S., Lee, S., Kim, Y., Lee, Y., Kang, M. W., Kim, K., Kim, Y. C., Han, S. S., Lee, H., Lee, J. P., Joo, K. W., Lim, C. S., Kim, Y. S., & Kim, D. K. (2021). Causal Effects of Positive Affect, Life Satisfaction, Depressive Symptoms, and Neuroticism on Kidney Function: A Mendelian Randomization Study. Journal of the American Society of Nephrology: JASN, 32(6), 1484–1496. https://doi.org/10.1681/ASN.2020071086

Rukh, G., Dang, J., Olivo, G., Ciuculete, D. M., Rask-Andersen, M., & Schiöth, H. B. (2020). Personality, lifestyle and job satisfaction: causal association between neuroticism and job satisfaction using Mendelian randomization in the UK Biobank cohort. Translational psychiatry, 10(1), 11. https://doi.org/10.1038/s41398-020-0691-3IF: 6.8 Q1

2. The elaboration of measurement methods of neuroticism would aid readers in better understanding the characteristics of neuroticism.

We appreciate your insightful suggestion.

Revision: Lines 69-72

In scientific research, personality measurement involving neuroticism is often operationalized and standardized by questionnaires, such as Eysenck Personality Questionnaire (Eysenck et al., 1985) and NEO Personality Inventory (Costa & McCrae, 1992), and Big Five Inventory (Soto & John, 2009).

Eysenck, S. B., Eysenck, H. J., & Barrett, P. 1985. A revised version of the psychoticism scale. Personality and individual differences, 6(1), 21-29.

Costa, P. T., & McCrae, R. R. 1992. Professional manual: revised NEO personality inventory (NEO-PI-R) and NEO five-factor inventory (NEO-FFI). Odessa, FL: Psychological Assessment Resources, 61.

Soto, C. J., & John, O. P. 2009. Ten facet scales for the Big Five Inventory: Convergence with NEO PI-R facets, self-peer agreement, and discriminant validity. Journal of research in personality, 43(1), 84-90.

3. In the introduction, regarding the association between postpartum depression and bipolar disorder, it would be helpful to elaborate on the potential biological mechanisms or clinical manifestations to deepen readers' understanding of this relationship.

We appreciate your insightful suggestion.

Revision: Lines 53-62

Neuroticism was linked to a single nucleotide polymorphism in the hydroxysteroid (11-beta) dehydrogenase 1 gene, mediating its association with postpartum depression (Iliadis et al., 2017). For maternal depression, expression levels of hydroxysteroid (11-beta) dehydrogenase positively correlate with serotonin transporter (Ponder et al., 2011). Serotonin transporter may influence the relationship between neuroticism and PPD (Canli & Lesch, 2007; Shapiro et al., 2012; Takano et al., 2007).

Iliadis, S. I., Comasco, E., Hellgren, C., Kollia, N., Sundström Poromaa, I., & Skalkidou, A. 2017. Associations between a polymorphism in the hydroxysteroid (11-beta) dehydrogenase 1 gene, neuroticism and postpartum depression. J Affect Disord, 207, 141-147. doi:10.1016/j.jad.2016.09.030

Ponder, K. L., Salisbury, A., McGonnigal, B., Laliberte, A., Lester, B., & Padbury, J. F. 2011. Maternal depression and anxiety are associated with altered gene expression in the human placenta without modification by antidepressant use: implications for fetal programming. Dev Psychobiol, 53(7), 711-723. doi:10.1002/dev.20549

Canli, T., & Lesch, K. P. 2007. Long story short: the serotonin transporter in emotion regulation and social cognition. Nat Neurosci, 10(9), 1103-1109. doi:10.1038/nn1964

Shapiro, G. D., Fraser, W. D., & Séguin, J. R. 2012. Emerging risk factors for postpartum depression: serotonin transporter genotype and omega-3 fatty acid status. Can J Psychiatry, 57(11), 704-712. doi:10.1177/070674371205701108

Takano, A., Arakawa, R., Hayashi, M., Takahashi, H., Ito, H., & Suhara, T. 2007. Relationship Between Neuroticism Personality Trait and Serotonin Transporter Binding. Biological Psychiatry, 62(6), 588-592.

4. In line 97, mentioning "to reduce sample overlap," it would be useful to further explain why sample overlap is a concern and the measures taken to ensure data independence and accuracy.

We appreciate the reviewer's comment regarding the need for further explanation regarding sample overlap in our manuscript. We re-evaluated the differences between single-sample and two-sample MR studies regarding the issue of sample overlap and listed the reasons most relevant to our study.

Revision: Lines 123-125

Due to the bidirectional causal testing design of the study, the genetic variant-exposure and genetic variant-outcome should come from different samples (Skrivankova et al., 2021).

Skrivankova, V. W., Richmond, R. C., Woolf, B. A. R., Yarmolinsky, J., Davies, N. M., Swanson, S. A., . . . Richards, J. B. 2021. Strengthening the Reporting of Observational Studies in Epidemiology Using Mendelian Randomization: The STROBE-MR Statement. Jama, 326(16), 1614-1621. doi:10.1001/jama.2021.18236

5. It would be advisable to list the references for choosing these specific thresholds in line 101-106.

Thanks for the reviewer's suggestion. We found literature that used the same threshold for screening as ours, which served as our basis.

Revision: Lines 135-137

These specific thresholds of summary-level statistics of the association of each genetic variant are required by Two-sample MR approach (Georgakis et al., 2019).

Georgakis, M. K., Gill, D., Rannikmäe, K., Traylor, M., Anderson, C. D., Lee, J. M., . . . Dichgans, M. 2019. Genetically Determined Levels of Circulating Cytokines and Risk of Stroke. Circulation, 139(2), 256-268. doi:10.1161/circulationaha.118.035905

6. In discussing the limitations of the study, the author mentioned the insufficient robustness of the IVW method, it is recommended that the author provide a more detailed explanation of the principles of these methods and how they impact the research results for a better understanding by the readers.

We appreciate the reviewer's valuable feedback. We explained the statistical limitations of other methods and demonstrated the robustness of the IVW method through the weighted median and leave-one-out approaches.

Revision: Lines 258-266

Once MR-Egger violates the no measurement error assumption, the bias it introduces tends to be greater than that of the IVW estimate, and it is particularly susceptible to the influence of weak instrument bias (Bowden, Del Greco, et al., 2016). The weighted mode method is sensitive for nonlinear effects, leading to biased results (Hartwig et al., 2017). However the weighted median method supports the robustness of IVW. In addition, we conducted outlier detection and removal strategies to outlier detection and removal strategies (Jack Bowden & Michael V. Holmes, 2019). Supplementary File 2 shows the good robustness of IVW.

Bowden, J., Del Greco, M. F., Minelli, C., Davey Smith, G., Sheehan, N. A., & Thompson, J. R. 2016. Assessing the suitability of summary data for two-sample Mendelian randomization analyses using MR-Egger regression: the role of the I2 statistic. Int J Epidemiol, 45(6), 1961-1974. doi:10.1093/ije/dyw220

Howard, L. M., Molyneaux, E., Dennis, C. L., Rochat, T., Stein, A., & Milgrom, J. 2014. Non-psychotic mental disorders in the perinatal period. Lancet, 384(9956), 1775-1788. doi:10.1016/s0140-6736(14)61276-9

Bowden, J., & Holmes, M. V. 2019. Meta-analysis and Mendelian randomization: A review. Research Synthesis Methods, 10(4), 486-496.

Reviewer #4 The study fills the rare literature on the biological linkage of personality as a predictor of mental health issues, especially for postpartum depression. The role of neuroticism as a risk factor of postpartum depression has been strongly reported in previous studies and this study has strengthened this inference using a robust causal analysis methodology. Therefore, the discussion section needs to explain more about the position of personality (neuroticism) in the context of genetic factors, emphasizing that this aspect of personality is not only a result of interaction of people with the environment or a social learning process but indeed is also a genetic factor. Referring to studies of the genetic role of personality in other context could also improve the significant role of genome to human behavior. This would allow the confident in the need of personality trait testing as has been highlighted in implication section.

We appreciate the reviewer's insightful comments and suggestions regarding the discussion section of our study. We acknowledge the importance of placing neuroticism within the context of genetic factors, particularly its role as a predictor of mental health issues such as postpartum depression, so we added a possible explanation for the position of personality (neuroticism) in the context of genetic factors. Please let us know if we should have any further changes.

Revision: Lines 219-227

The genetic loci associated with neuroticism contain genes related to depression, primarily distributed on chromosomes 2 and 19 genetic loci (Nagel et al., 2018). Extensive functional annotations of neuroticism include the neurogenesis pathway, which would be impaired by Postpartum estrogen withdrawal (Nagel et al., 2018; Zhang et al., 2016). Meanwhile, the neural circuit activity of GABAergic neurons projecting to serotonergic neurons may decrease due to ovarian hormone withdrawal, leading to the occurrence of depression, while serotonergic neurons are involved in the genetic correlation of neuroticism, implicating the involvement of specific cell types (Nagel et al., 2018; Tao et al., 2023).

Nagel, M., Jansen, P. R., Stringer, S., Watanabe, K., de Leeuw, C. A., Bryois, J., . . . Posthuma, D. 2018. Meta-analysis of genome-wide association studies for neuroticism in 449,484 individuals identifies novel genetic loci and pathways. Nat Genet, 50(7), 920-927. doi:10.1038/s41588-018-0151-7

Zhang, Z., Hong, J., Zhang, S., Zhang, T., Sha, S., Yang, R., . . . Chen, L. 2016. Postpartum estrogen withdrawal impairs hippocampal neurogenesis and causes depression- and anxiety-like behaviors in mice. Psychoneuroendocrinology, 66, 138-149. doi:10.1016/j.psyneuen.2016.01.013

Tao, C., Zhang, G. W., Huang, J. J., Li, Z., Tao, H. W., & Zhang, L. I. 2023. The medial preoptic area mediates depressive-like behaviors induced by ovarian hormone withdrawal through distinct GABAergic projections. Nat Neurosci, 26(9), 1529-1540. doi:10.1038/s41593-023-01397-2
